# Leucokinin and Associated Neuropeptides Regulate Multiple Aspects of Physiology and Behavior in *Drosophila*

**DOI:** 10.3390/ijms22041940

**Published:** 2021-02-16

**Authors:** Dick R. Nässel

**Affiliations:** Department of Zoology, Stockholm University, S-10691 Stockholm, Sweden; dnassel@zoologi.su.se

**Keywords:** diuretic hormone, sleep, feeding, metabolism, ion transport peptide, tachykinin, short neuropeptide F, insulin-like peptide, neuromodulation

## Abstract

Leucokinins (LKs) constitute a family of neuropeptides identified in numerous insects and many other invertebrates. LKs act on G-protein-coupled receptors that display only distant relations to other known receptors. In adult *Drosophila*, 26 neurons/neurosecretory cells of three main types express LK. The four brain interneurons are of two types, and these are implicated in several important functions in the fly’s behavior and physiology, including feeding, sleep–metabolism interactions, state-dependent memory formation, as well as modulation of gustatory sensitivity and nociception. The 22 neurosecretory cells (abdominal LK neurons, ABLKs) of the abdominal neuromeres co-express LK and a diuretic hormone (DH44), and together, these regulate water and ion homeostasis and associated stress as well as food intake. In *Drosophila* larvae, LK neurons modulate locomotion, escape responses and aspects of ecdysis behavior. A set of lateral neurosecretory cells, ALKs (anterior LK neurons), in the brain express LK in larvae, but inconsistently so in adults. These ALKs co-express three other neuropeptides and regulate water and ion homeostasis, feeding, and drinking, but the specific role of LK is not yet known. This review summarizes *Drosophila* data on embryonic lineages of LK neurons, functional roles of individual LK neuron types, interactions with other peptidergic systems, and orchestrating functions of LK.

## 1. Introduction

A large number of structurally diverse neuropeptides have been identified in insects, and they are known to act on different types of receptors, predominantly G protein-coupled receptors (GPCRs), as co-transmitters, neuromodulators and hormones [1,2,3,4]. Neuropeptides are major players in regulation of development, growth, reproduction, physiology and behavior and they are, thus, important throughout the lifecycle [1,2,5,6,7,8,9]. Insects have, for a long time, served in analysis of neuropeptide signaling and endocrinology [10,11,12]. The first insect neuropeptide to be isolated and sequenced was proctolin in 1975 [13], and a first neuropeptide GPCR (CG7887, Takr99D) was cloned in *Drosophila* in 1991 and found to be an ortholog of mammalian tachykinin receptors [14]. Today, more than 50 neuropeptide precursors and corresponding receptors are known in *Drosophila* and a large number of other insects, and much progress has been made in understanding the complexity of neuropeptide signaling [1,2,15,16,17]. *Drosophila* has emerged as a versatile model organism in studies of peptide signaling due to the extensive genetic toolbox available, together with access to a huge number of transgene flies from stock centers and the fly community, resources for functional genomics, functional imaging techniques, advances in neuronal connectomics, single-cell transcriptomics and development of efficient physiological and behavioral assays (see, e.g., [18,19,20,21,22,23,24]). So far, some peptide systems in *Drosophila* have attracted substantial attention, whereas others are largely neglected (see [1,5,7,25]). One of the neuropeptides that has gained traction recently in *Drosophila* is leucokinin (LK). Thus, it is timely to review what is known about LK signaling in *Drosophila*.

LKs were first identified from the Madeira cockroach *Leucophaea maderae* (now *Rhyparobia maderae*) in 1986 by assaying purified fractions for myostimulatory activity (see [26,27,28]). In this cockroach, eight LKs (sequence-related paracopies) were identified that are characterized by the C-terminus FXSWGamide [28,29]. *Drosophila* LK was identified much later and has the full sequence NSVVLGKKQRFHSWGamide [30]. It should be emphasized at the outset that LKs and their receptors are not related to tachykinins and their receptors [15,16,31], although this was suggested earlier on. In fact, LK signaling appears restricted to a few invertebrate phyla only [15,16].

In addition to stimulatory action on visceral muscle of some insects, LKs were found to act *ex vivo* as a factor that increases secretion in the Malpighian (renal) tubules of various insects [30,32,33,34,35]. For many years, the diuretic and myotropic actions of LKs were the only ones known. However, when appropriate genetic tools became available for *Drosophila*, a second wave of investigations of LK functions was initiated. Thus, over recent years, an impressive array of functional roles of *Drosophila* LK signaling have been uncovered. Furthermore, as we shall see in this review, it has been possible to pinpoint the functional roles of individual LK-expressing neuron types.

One remarkable feature of the LK system in *Drosophila* is that the peptide is expressed consistently in only 26 neurons of three major types in the adult central nervous system (CNS). Four of these LK cells are interneurons in the brain and subesophageal zone (SEZ); the remaining ones are segmentally arranged neurosecretory cells in the abdominal neuromeres of the ventral nerve cord (VNC) [36,37,38,39]. Yet the two pairs of brain/SEZ neurons are the basis for a wide range of modulatory and orchestrating actions in fly physiology and behavior. Furthermore, LK plays developmental roles during ecdysis and acts as a neuromodulator of larval locomotion [40,41,42]. In addition to the two pairs of relatively well-characterized LK neurons in the brain/SEZ, there is also a set of four pairs of brain neurons (ALKs) (anterior LK neurons; anterior in larval brain-body axis, but posterior in adult brain due to morphogenetic changes during metamorphosis) that are consistently labeled with different *Lk*-Gal4 lines but display variable LK immunolabeling [36,38]. These ALKs are lateral neurosecretory cells that co-express tachykinin (TK), short neuropeptide F (sNPF) and ion transport peptide (ITP) [43,44], but the functional role of LK in these neurons is not yet known. However, some functions of the other three peptides in the ALKs have been revealed [43,45] and will be discussed below.

In *Drosophila*, the gene encoding the LK precursor (CG13480) gives rise to a single copy of the peptide and only one LK receptor (LKR; CG10626) is known, which facilitates studies [3,4,30,46]. The neurons producing LK have been described in detail anatomically, whereas the functional LKR distribution is only partly known [36,37,38,39,46,47,48,49]. This review summarizes different aspects of LK signaling in *Drosophila* with a special focus on the role of individual types of LK-producing neurons and the circuits in which they function together with other neuropeptides. Furthermore, the embryonic lineages of LK neurons are discussed. Taken together, the LK neurons regulate water and ion homeostasis and associated stress responses, feeding, sleep–metabolism interactions, state-dependent memory formation, as well as modulation of gustatory sensitivity, nociception and post-mating behavior. It is noteworthy that LK signaling seems absent in many invertebrate taxa as well as in vertebrates, and yet, this peptide serves such vital functions in *Drosophila*.

## 2. Distribution of LK and Its Receptor in *Drosophila*

In this section, advancements in understanding the functional roles of LK-expressing neurons in *Drosophila* are discussed. In addition to the powerful genetic methods available, *Drosophila* shares with many invertebrates the advantage of having a relatively small number of neurons, many of which can be individually identified. Thus, it is possible to assign neuropeptides and other neuromodulators/transmitters as well as functions to single neurons that can be repeatedly assayed in multiple fly specimens. This, together with fly connectomics data [50,51,52,53] and single-cell transcriptomics [21,24], means that we should eventually be able to obtain a very good handle on the roles of neuropeptide signaling in brain circuitry.

### 2.1. The Power of Identifiable Neurons in Studies of Neuropeptide Signaling

Brains of annelids, arthropods and mollusks are remarkable in that they contain many unique neurons and neurosecretory cells that are specified by their location, size, anatomy and connections as well as their neurotransmitter and/or neuromodulator expression (see [54,55,56,57]). A first example of large, unique neurons was described already in 1891, the so-called Retzius cells of the leech [58,59]. Such neurons can be identified in every specimen (often even in related species) by the consistent location of their cell bodies and are referred to as identifiable neurons. In organisms such as mollusks, the identified neurons were the objects of intense research earlier on due to their large size and, thus, the ease by which electrophysiological recordings could be made (see [56,60,61]). It was even possible to use extracts of single identified cells to perform biochemical identification of neurotransmitters and neuropeptides, or cloning of neuropeptide precursor cDNA [60,62]. Thus, mollusks such as *Aplysia californica* and *Lymnaea stagnalis* were at the forefront in analysis of invertebrate neurotransmission, neuromodulation and peptide function earlier on (see [60,61,63,64,65,66]). Early progress was also made in decapod crustacean models using the stomatogastric ganglion (STG) with a small number of large neurons whose connectivity had been established already in the 1970s ([67,68], and summarized in [69,70,71]). A repertoire of neurotransmitters and neuromodulators in identified neurons in the STG (and associated ganglia) was established by immunohistochemistry and network analysis performed with a combination of electrophysiology and application of neurotransmitters/peptides (see [71]). In comparison, progress in peptidergic neuromodulation in insects was slower, since most systems under study were more complex anatomically, and the neurons were smaller and not as accessible to the techniques available at the time. Some insect neurons, such as dorsal unpaired median (DUM) neurons, brain descending neurons and specific large motoneurons, were, however, explored by electrophysiology to understand neuromodulation and generation of motor activity (see [72]).

A breakthrough for insect neuropeptide studies was the advent of novel *Drosophila* methods, such as the Gal4-UAS technique, that made it possible to target single genes encoding neuropeptides and their receptors or target identified neurons to enable anatomical analysis or interference with neuron function [73,74,75]. For example, by expressing apoptosis genes (reaper, *rpr*, or head involution defective, *hid*), the single pair of neurons producing eclosion hormone (EH) could be eliminated and the effects on fly eclosion analyzed [76], or a small set of peptidergic clock neurons, expressing pigment-dispersing factor, were ablated to investigate effects on the daily rhythmicity of the flies [77]. These methods have, over recent years, been much refined so that it is possible not only to target genes encoding specific neuropeptides and their receptors, or neurons expressing these, but also to do so conditionally at specific times in the lifecycle of *Drosophila* [78]. Intersectional genetic techniques have made it possible to target subpopulations of neurons expressing specific genes, and optogenetic and thermogenetic activation of specific peptidergic neurons can be performed [78,79,80]. Different reporter systems enable functional imaging (see [78,80,81]) and establishment of synaptic connections between identified neurons [82,83,84]. Thus, today, we can dissect the functional roles of neuropeptides in single cell types, specific neuronal circuits or in cells of other tissues by a large set of techniques. This has been important in understanding that neuropeptides and peptide hormones are pleiotropic and that their roles can be different at different times in the lifecycle, or at different sites in the brain (see [7,85]).

Some peptides appear to be utilized by neurons (and other cells) to globally orchestrate development, physiology or behavior, whereas others seem to play multiple distributed roles that are more localized and circuit-specific [85]. The action of the latter type of peptides may be in the form of co-transmission together with other neurotransmitters and neuromodulators [71,86,87]. Taken together, these findings have led to the insight that certain neuropeptides/peptide hormones can be remarkably diverse functionally, although they were initially identified from tissue extracts in a single bioassay (see [1]). Certain peptides such as tachykinins (TK) and short neuropeptide F (sNPF) are produced by large numbers of diverse neurons and appear truly multifunctional, where peptide actions are highly localized and, therefore, function is dependent on the circuits where TK or sNPF receptors are activated [31,85,88]. Other peptides such as LKs are produced by a small set of neurons and neurosecretory cells [36,89]. Thus, peptide action from these LK neurons can be both local and hormonal and appears to be orchestrating physiology and behavior at the organism level [38,48]. As we shall see next, in *Drosophila*, single LK neuron types can use this neuropeptide for multiple purposes, sometimes together with colocalized neuropeptides. However, it is likely that the different LK neurons are functionally coordinated and that the peptide modulates and orchestrates the physiology and behavior of the fly in a state-dependent fashion.

### 2.2. Localization of LK and LKR in Drosophila and Colocalization with Other Neuropeptides

**Figure 1:**
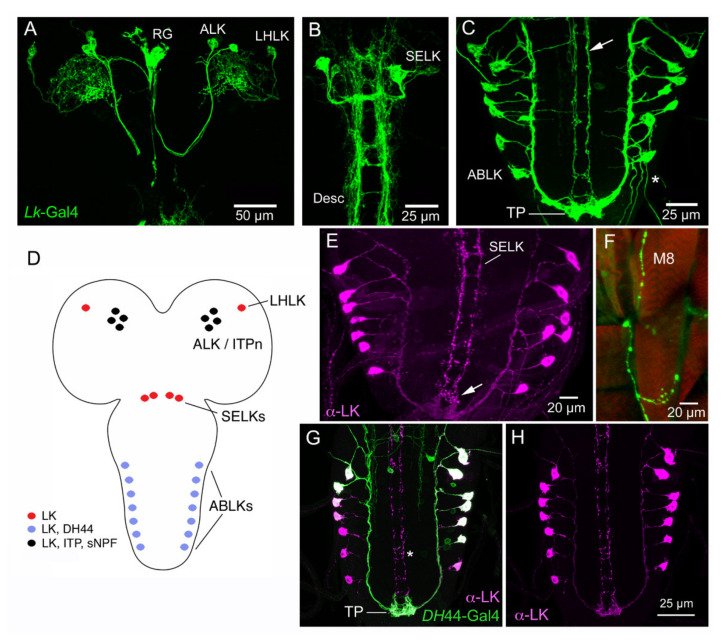
Expression of leucokinin (LK) and other peptides in the CNS of larval *Drosophila*. (**A**) Larval brain with *Lk*-Gal4 expression in LHLK (lateral horn LK) and ALK (anterior LK) neurons. The ALKs have axon terminations in the ring gland (RG). (**B**) SELK (subesophageal LK) neurons of the larval brain/ventral nerve cord with their descending axons (Desc). (**C**) The abdominal leucokinin neurons (ABLKs) and axons of SELKs (arrow). Note the terminal plexus neuropil (TP) with axonal processes of both ABLKs and SELKs. (**D**) Schematic of the LK-expressing cell bodies. Colors indicate the different peptides expressed in the neurons. (**E**) LK immunolabeling of ABLKs and SELK axons. The arrow indicates the terminal plexus. (**F**) Axon termination of an ABLK on body wall muscle 8 (M8). (**G**,**H**) The ABLKs, but not SELKs (asterisk), co-express LK and diuretic hormone 44 (DH44). Images in Figure 1 are modified and rearranged from [37,96], with permission from publishers.

**Figure 2:**
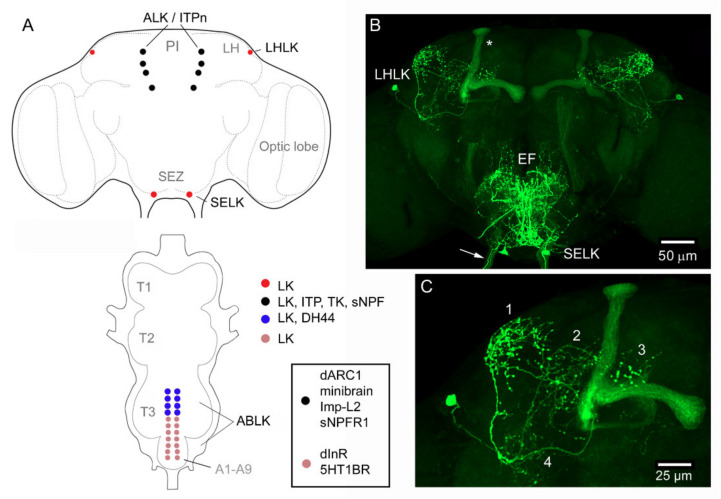
*Lk*-Gal4-expressing neurons in the adult *Drosophila* CNS. (**A**) Schematic of brain and ventral nerve cord (VNC) with distribution of LK-expressing neuronal cell bodies. The box shows expression of other proteins in two sets of LK cell bodies: dARC1, activity-regulated cytoskeleton-associated protein [97]; Imp-L2, Imaginal morphogenesis protein-Late 2 [98]; sNPFR1, short neuropeptide F receptor 1 [99]; dInR, insulin receptor [100]; 5HT1BR, serotonin receptor 1B [100]. The ALK/ITPn (LK-ITP expressing neurons) also express the leucokinin receptor (LKR), but no function has been established so far. Other abbreviations: PI, pars intercerebralis; LH, lateral horn; SEZ, subesophageal zone; T1–T3, thoracic neuromeres; A1–A9, abdominal neuromeres. (**B**) The two LK-expressing neuron types in the brain. Arrows indicate axons in a subesophageal nerve; asterisk indicates lobes of the mushroom body expressing GFP. EF, esophageal foramen. (**C**) Enlarged image of one LHLK neuron with four major areas of arborizations indicated by numbers. Images in Figure 2 are modified and rearranged from [37,38], with permission from publishers.

*Drosophila* LK neurons were first described in the larval nervous system [89] and later in the adult fly [90,91]. In the larval CNS, antisera to LK identify 20 LK neurons: a pair of LHLKs (lateral horn LK neurons) in the lateral horn of the brain, two pairs of SELKs (subesophageal LK neurons) in the subesophageal zone (SEZ) and seven pairs of abdominal leucokinin neurons (ABLKs) in abdominal neuromeres A1–A7 (Figure 1), whereas in the adult, one pair of SELKs is lost and three to four pairs of ABLKs have been added during pupal development [36] (Figure 2). The ABLKs send varicose axons to body wall muscles (muscle 8 in larvae) and the lateral heart nerve, as well as to spiracles [36,89]. It can be presumed that these sites serve as neurohemal release sites for hormonal LK. Circulating LK has not been demonstrated in *Drosophila*. However, in some insects, LK is produced by neurosecretory cells that have axon terminations in the corpora cardiaca (CC), and a calcium-dependent, potassium-induced release of LKs from the CC (*ex vivo*) of *L. maderae* [92] and *Acheta domesticus* [93] has been demonstrated. Taken together with the demonstration of circulating LK in the hemolymph after feeding in the bloodsucking bug *Rhodnius prolixus* [94], there is, thus, support for hormonal roles of LKs. Hormonal LK action in *Drosophila* is further suggested since the Malpighian tubules, which respond to LK *ex vivo* [30,46], are not innervated but rather freely exposed to the circulation, and it was shown that knockdown of the LKR in the periphery, but not in the CNS, affects water and ion homeostasis [95].

With the use of *Lk*-Gal4 drivers, the immunocytochemistry findings have been confirmed and extended [36,37]. Gal4 expression revealed further details of the anatomy of LK neurons. For instance, it can be seen that the SELKs have axonal processes descending into the ventral nerve cord [36,38] (see Figure 1). The LHLKs have very profuse arborizations, not only in the lateral horn but also in other neuropils of the dorsolateral protocerebrum (Figure 2). Furthermore, *Lk*-Gal4 expression can additionally be seen in four pairs of posteriorly located protocerebral neurosecretory cells (ALKs) in the adult, and three to four pairs in the larval brain [36] (Figure 1). In the early larva, these cells also label with LK antisera, whereas in adults, labeling is variable [36,38]. It was found that the ALKs are identical to sets of neurons that express ion transport peptide (ITP), sNPF and TK [37,43]. These ITP-producing neurons (also designated ITPn) are lateral neurosecretory cells (LNCs) that have axon terminations in the corpora cardiaca, corpora allata and anterior aorta as well as branches in the SEZ and tritocerebrum [43,44]. These neurons are henceforth designated ALK/ITPn. Occasionally, one can also see LK immunolabeling in the adult ALK/ITPn cells [38], suggesting that these neurons can generate products of four different neuropeptide precursors. It is, however, not clear at present under which conditions LK is upregulated in the adult ALK/ITPn. I shall return to the ALK/ITPn as well as the other LK neurons and their functions later on.

The ABLKs in *Drosophila* co-express diuretic hormone 44 (DH44) [37] (Figure 1 and Figure 2). This was also found to be the case for homolog cells in some other insects such as the housefly *Musca domestica*, the blood-sucking bug *Rhodnius prolixus*, locust *Locusta migratoria* and moth *Manduca sexta* [101,102,103,104]. In the *Drosophila* larva, all ABLKs express DH44, whereas in the adult, the strongest DH44 expression is seen in the 6–8 anterior adult-specific ABLKs, and more variable expression was noted in the 14 embryo-derived cells [37]. Thus, out of four major LK neuron types, two (ALK/ITPn and ABLK) co-express other peptides. In Table 1**,** genes/proteins of interest expressed in LK neurons of *Drosophila* are shown. The transcription factor Dimmed (Dimm) [105] can be detected in LHLKs, ALK/ITPn and ABLKs, but not in SELKs [106,107]. Dimmed is known to confer a neurosecretory phenotype to peptidergic neurons by increasing their capacity for production and storage of peptides to enable intermittent bulk release [105,107,108]. Thus, it is somewhat surprising that the large SELKs with descending axons do not express detectable Dimm.

**Figure 3:**
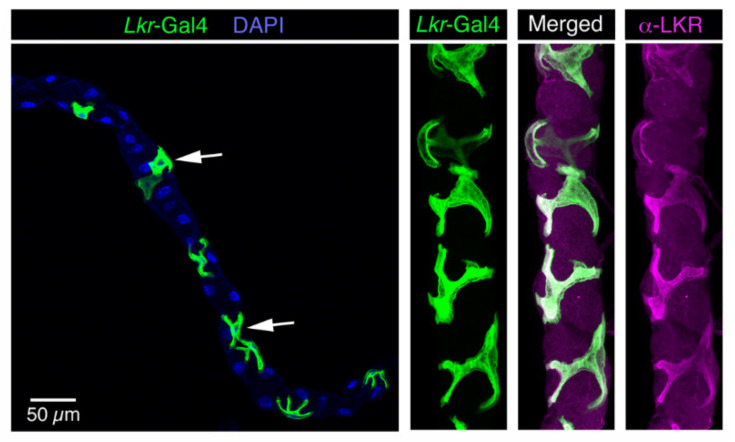
Expression of leucokinin receptor Gal4 (*Lkr*-Gal4) in stellate cells of the Malpighian tubules of adult *Drosophila*. Arrows indicate stellate cells. Nuclei are stained with DAPI (blue). The stellate cells also label with antiserum to the *Drosophila* LKR (shown in magenta). Figure reproduced from [38] with permission from the publisher.

**Figure 4:**
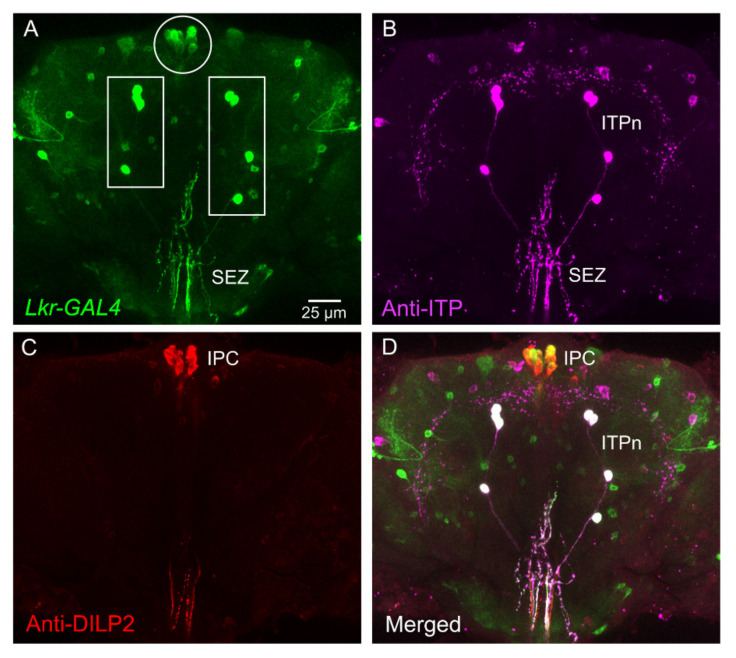
Expression of leucokinin receptor (*Lkr*) in identified neurons of the adult *Drosophila* brain. (**A**) *Lkr*-Gal4 expression in the brain and subesophageal zone (SEZ). The boxed areas contain ALKs (ALK/ITPn) and the encircled area shows the insulin-producing cells (IPCs). (**B**) Ion transport peptide (ITP) immunolabeling in the same brain. The ITPn that also express LK are indicated. (**C**) DILP2 (insulin-like peptide 2) immunolabeling in IPCs in the same brain. (**D**) Merging the three channels, it is clear that ITPn and IPC co-express *Lkr*-Gal4 (these are boxed and encircled in A, respectively). Figure reproduced from [38] with permission from the publishers.

**Figure 5:**
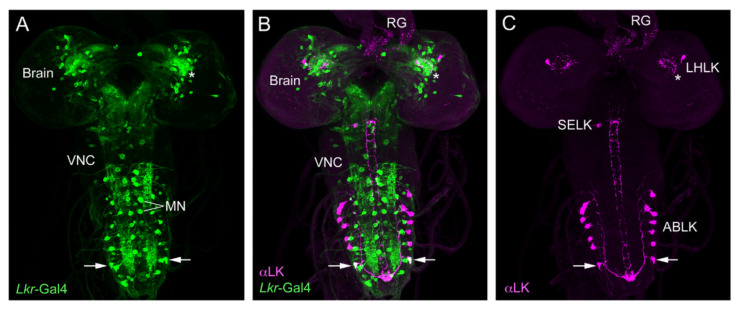
Expression of LK immunolabeling and *Lkr*-Gal4 in the larval CNS. (**A**) *Lkr*-Gal4 is seen in numerous neurons throughout the CNS. Some are large segmental motoneurons (MN) of the abdominal neuromeres known to innervate body wall muscle (either aCC or RP2 motoneurons). VNC, ventral nerve cord. (**B**) LK immunolabeling and *Lkr*-Gal4 reveals that one pair of ABLKs display *Lkr*-Gal4 expression (arrows). Note the strong expression of *Lkr* in the lateral horn (asterisk) where LHLKs arborize. (**C**) LK immunolabeled neurons. The axon terminations in the ring gland (RG) are from ALK neurons (their cell bodies are not seen in this focal plane). Figure reproduced from [38] with permission from the publisher.

**Figure 6:**
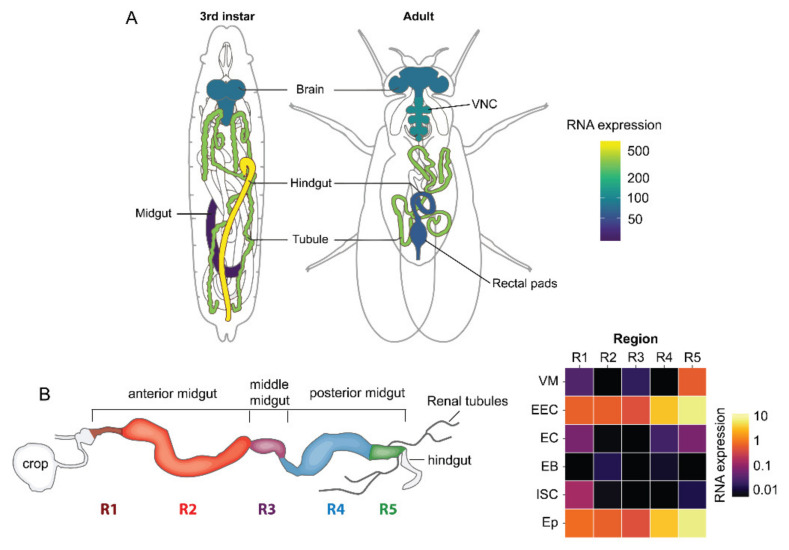
Expression of the leucokinin receptor (*Lkr*) in tissues of *Drosophila*. (**A**) Schematics of third instar larvae and adult fly showing the expression of *Lkr* (Data from FlyAtlas.org, [112]). (**B**) A schematic of the adult intestine and heat map showing expression of *Lkr* in different regions of the gut (R1 to R5) and its various cell types (VM, visceral muscle; EEC, enteroendocrine cell; EC, enterocyte; EB, enteroblast; ISC, intestinal stem cell; Ep, epithelium). Data were mined using Flygut-seq [113]. This figure was reproduced from [38] with permission from the publisher.

Antiserum to the LK receptor (LKR) was used to demonstrate expression in stellate cells of the Malpighian tubules and some neurons in the brain [46]. The same laboratory later employed fluorophore-tagged LK to localize ligand binding to the stellate cells and some neurons in the larval CNS [109]. However, more detailed mapping of LKR distribution in the CNS has relied on the expression of different *Lkr*-Gal4 lines [38,39,47,48]. LKR expression in stellate cells was confirmed (Figure 3) and it was found that there is *Lkr* expression in the insulin-producing cells (IPCs) of the brain, in the ALK/ITPn, in a few larval ABLKs and numerous unidentified neurons [38] (Figure 4 and Figure 5). Other studies demonstrated *Lkr*-expression in neurons innervating the upper layer of the fan-shaped body of the central complex [39,47,110]. Further *Lkr*-Gal4 expression was shown in muscle fibers of the anterior midgut and posterior hindgut, as well as in enteroendocrine cells of the midgut and in structures of the rectal pad [38] (Figure 6). Functional expression of the LKR has, so far, been confirmed experimentally only for stellate cells and IPCs [30,38,46,48]. Thus, further efforts are required to map the distribution of the LKR in *Drosophila* in more detail and to validate functional expression. It is especially intriguing that the LK-expressing ALK/ITPn also express the LKR, and this suggests that neurons such as SELKs might provide inputs to those neurosecretory cells.

**Table 1:** Genes/proteins of interest that are expressed in LK neurons in *Drosophila.*

Cell Type	Co-Expression	Stage	Function	References
LHLK	Translin	Adult	Starvation-induced sleep suppression	[111]
LHLK	Dimmed	Larva, adult	Neuron size, LK production	[106]
LHLK	AMPK ^1^	Adult	Increases LHLK activity in fed flies	[48]
SELK	dInR	Adult	Not tested	[96]
ALK/ITPn	ITP, sNPF, TK	Larva ^2^, adult	Stress responses, water and ion homeostasis, drinking and feeding^3^	[43,45]
ALK/ITPn	LKR	Adult	Not tested	[38]
ALK/ITPn	ImpL2 ^4^	Adult	Promotes insulin signaling and uptake	[98]
ALK/ITPn	dInR	Adult	Activates insulin signaling	[98]
ALK/ITPn	dARC1 ^5^		Starvation resistance, starvation-induced hyperactivity	[97]
ALK/ITPn	Minibrain	Adult	Regulates food intake and sNPF expression	[99]
ALK/ITPn	sNPFR1	Adult	Regulates food intake	[99]
ALK/ITPn	Dimmed	Adult	Neuron size	[106]
ABLK	DH44	Larva, adult	Water and ion homeostasis ^2^	[37]
ABLK	5HT1B-R ^6^	Adult	Modulates desiccation response	[100]
ABLK	5HT1B-R ^6^	Larva	Larval locomotion	[42]
ABLK	dInR	Larva, adult	Neuron size, LK production	[96,100]
ABLK	LKR	Larva ^7^	Not tested	[38]
ABLK	Dimmed	Larva, adult	Neuron size, LK production	[106]
ABLK	ETH-R ^7^	Larva	Pre-ecdysis behavior	[40]
ABLK	ETH-R	Larva	Tracheal clearance	[41]

Notes: ^1^ AMPK, 5′ adenosine monophosphate-activated protein kinase. ^2^ TK not in larval ALK/ITPn. ^3^ Only tested in adults. ^4^ ImpL2, Imaginal morphogenesis protein-Late 2 (Ecdysone-inducible gene L2). ^5^ dARC1, activity-regulated cytoskeleton-associated protein (ARC)—also expressed in IPCs and AKH cells (APCs). Similar to ImpL2. ^6^ 5HT1B-R, serotonin receptor 1B. ^7^ Only the larva was analyzed, and only one pair of ABLKs express dInR (Figure 5A–C). ^8^ ETH-R, Ecdysis-triggering hormone receptor.

**Figure 7:**
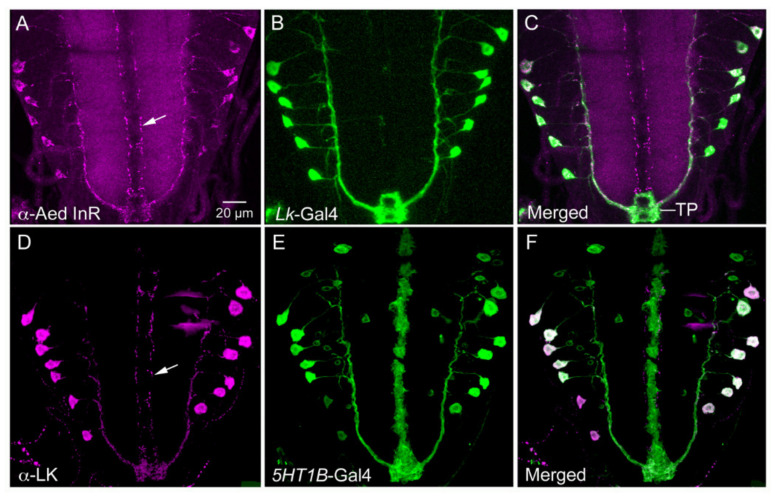
LK-expressing neurons, ABLKs, in the *Drosophila* larva co-express receptors of insulin and serotonin. (**A**–**C**) Antiserum to *Aedes aegypti* insulin receptor (Aed InR) labels *Lk*-Gal4 expressing ABLKs and axons of SELKs (arrow). (**D**–**F**) LK antiserum labels ABLKs that also express 5HT1B-Gal4 (serotonin receptor 1B). Note that SELK axons (arrow) do not express 5HT1B receptor Gal4. A is from [96] and B from [100], with permission from the publishers.

Some of the genes/proteins co-expressed in LK neurons (Table 1) were discussed above and others are dealt with in the next section. It is noteworthy that a few neurotransmitter/neuropeptide receptors were shown to be functionally expressed by subsets of the LK neurons: the insulin receptor dInR (Figure 7A–C), the serotonin receptor 5-HT1B (Figure 7D,F), the sNPF receptor (sNPFR1) and the ecdysis-triggering hormone (ETH) receptor [40,41,99,100], and more are likely to be identified from single-cell transcriptome studies.

## 3. Functions of Specific LK-Expressing Neurons in *Drosophila*

Earlier studies of LK in *Drosophila* did not analyze the functions of specific cell types, although it was assumed that ABLKs are involved in systemic hormonal regulations since they are the only neurosecretory cells consistently expressing high levels of LK in the adult fly [36,89,100]. Thus, it was assumed that the phenotypes in water and ion homeostasis, abdominal bloating and desiccation responses seen after global *Lk*-RNAi (RNA interference) or silencing or LK neurons were mediated mainly by ABLKs [100,114]. Manipulation of activity in all LK neurons also affected the composition of fecal deposits and rate of defecation in flies [95]. In support of a hormonal role of LK, the same study showed that LKR in the intestine and renal tubules, but not in the CNS, is necessary for phenotypes associated with water and ion homeostasis and defecation [95]. As shown in Table 2, similar global interventions also affected chemosensory responses, feeding behavior and lifespan [39,100,114]. More specifically, *Lk* and *Lkr* mutant flies displayed increased crop size and bloated abdomens as well as altered meal sizes in flies starved previously [39]. The bloated abdomens were later found to be associated with dysregulated diuresis, likely due to ABLKs [95,100], but the alteration of food intake pattern and crop size may be due to brain neurons.

### 3.1. Functions of ABLKs

ABLKs, but no other LK neurons, were found to co-express DH44 and a serotonin receptor (5HT1B) (Figure 1D–H and Figure 7D–F; Table 1), and that allowed for specific manipulations of these cells [37,100]. Knockdown of LK only in ABLKs using *DH44*-Gal4 resulted in effects on water retention, desiccation and ionic stress, suggesting that these neurons are sufficient for the effect on the renal tubules and intestine [37]. Application of serotonin to abdominal neuromeres diminishes spontaneous calcium activity in ABLKs, and knockdown of the 5-HT1B receptor in ABLKs results in diminished LK expression in ABLKs and increased desiccation resistance, indicating that modulated activity in ABLKs affects water balance [100]. Further experiments support a role for ABLKs in water and ion homeostasis [38]. The DH44 co-expressed in ABLKs also contributes to diuretic functions and, additionally, to regulation of food intake [37]. Knockdown of the 5HT1B receptor in ABLKs also diminished food intake [100]. In larval *Drosophila,* ABLKs are involved in the regulation of locomotor turning behavior [42], pre-ecdysis behavior [40] and tracheal clearance at ecdysis [41]. The role of insulin receptor expression in ABLKs appear to be in regulation of LK expression and as a regulator of neuron size during development [96,100]. The input neurons that act on the receptors found on ABLKs (Table 1) have not yet been identified; however, SELKs may act on these neurons, at least in larvae, since the LKR is expressed by a minimum of one pair or ABLKs, and the two neuron types are in close contact posteriorly in the VNC (Figure 5A–C) (see also [38]).

**Figure 8:**
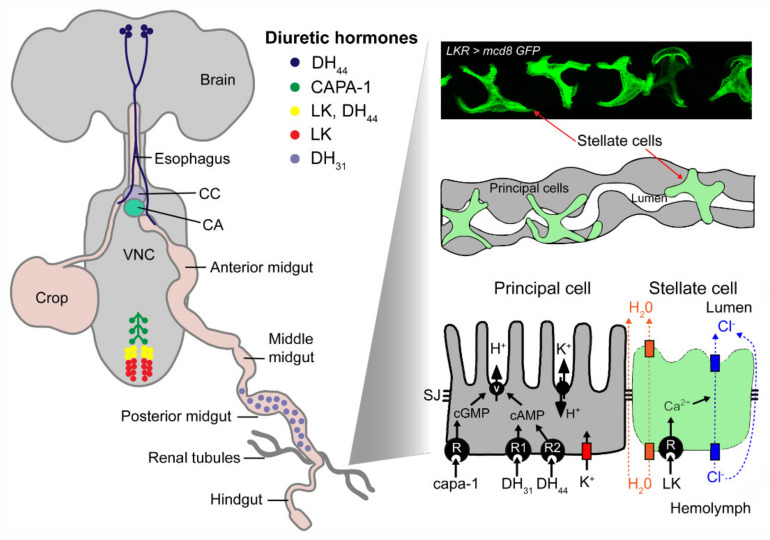
Distribution and actions of LK and other diuretic hormones in adult *Drosophila*. A schematic depiction of the location of peptidergic neurons and gut endocrine cells that express diuretic hormones: *capability*-derived peptides (CAPA1-2), diuretic hormone 31 (DH31), diuretic hormone 44 (DH44) and leucokinin (LK). After release from these neurosecretory cells, the peptide hormones act on their receptors that are localized in either of two major cell types in the Malpighian (rental) tubules, the principal cells or stellate cells (visualized here using *Lkr* > mcd8GFP). The peptides act via different second messenger systems to alter the activity of ion pumps or channels. The orange rectangles represent aquaporin channels, the blue represent chloride channels and the red represents a Kir potassium channel. Abbreviations: CC, corpora cardiaca; CA, corpora allata; VNC, ventral nerve cord; SJ, septate junction; V, V-type ATPase. Figure 8 is based on a figure from [1]. The Malpighian tubule cell model is adapted and redrawn from O’Donnell et al. [131]. The image of localization of LKR in stellate cells is from Zandawala et al. [37] with permission from the publishers.

ABLKs constitute one of several sets of peptidergic neurons that regulate ion and water balance (Figure 8, Supplementary Figure S1). Other peptides known to act as diuretic and antidiuretic factors are DH44, DH31, capability (CAPA1-2) peptides, ITP and maybe glycoprotein A2/B5 (GPA2/GPB5) [45,121,122,123,124,125,126,127,128], whereas pigment-dispersing factor (PDF) acts on muscle contractions in the ureter portion of the renal tubules of adults [129] and hindgut in larvae [130]. Global knockdown of GPB5 did not affect desiccation or regulate ionic stress, only resistance to starvation [128]. Thus, the eight pairs of neurosecretory cells in the A1–A4 neuromeres that express GPB5 may act in different aspects of diuresis/antidiuresis.

The mechanisms of peptide action on renal tubules have been studied. LK stimulates intracellular calcium in stellate cells of the renal tubules and thereby promotes chloride conductance and water transport through the epithelium [132] (Figure 8). The two hormones DH31 and DH44 act on the principal cells to activate cAMP and V-ATPase and, hence, secretion [122,125], whereas CAPA peptides act on the same cells but by activation of intracellular calcium, nitric oxide and cGMP signaling [124,126] (Figure 8, Supplementary Figure S1). Thus, ion and water homeostasis is tightly regulated and it remains to be clarified how the different hormonal systems cooperate in daily life and how internal sensors that monitor the osmotic state regulate the neurosecretory cells that release diuretic/antidiuretic hormones [133].

**Figure 9:**
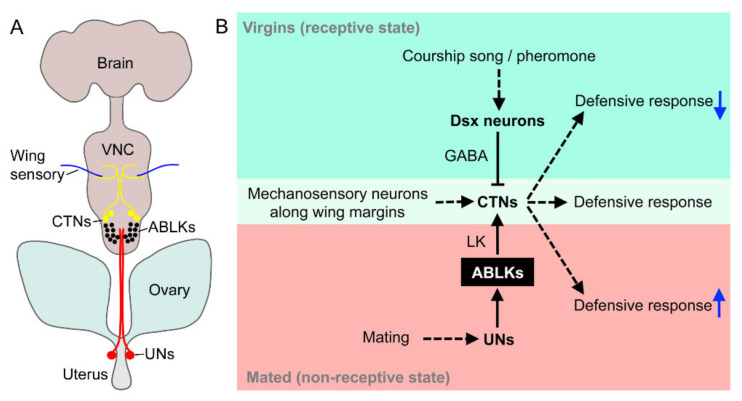
The role of LK signaling and ABLKs in a post-mating defensive response. (**A**) Pathway that regulates a defensive wing retraction response in female flies [118]. Mechanosensory cells in the wing margin send axons to the mesothoracic neuromere of the ventral nerve cord (VNC). They synapse on interneurons (CTNs) that modulate motoneurons, which retract the wings (not shown). A set of sensory cells (UNs) in the uterus synapse with the ABLKs. After mating, these UNs are stimulated by contents (not sex peptide) in the semen and activate the ABLKs. The ABLKs in turn activate the CTNs, and the defensive response is strengthened. (**B**) Diagram of the circuit regulating the defensive response. Virgin female flies do not display this wing defense response. This is ensured by a pathway where sensory inputs (courtship song and pheromones) to female-specific neurons that express the gene *doublesex* (Dsx) use GABA (γ-aminobutyric acid) to inhibit the CTNs. In mated flies, the UNs in the uterus activate ABLKs and the CTNs enhance the defensive response. Figure 9A was drawn based on data in [118] and Figure 9B was redrawn from a figure in [118].

A recent paper described a very different and somewhat surprising role of ABLKs in female *Drosophila* [118]. The ABLKs in adult female flies serve as an interface between sensory neurons of the oviduct and interneurons in the thoracic ganglia that regulates a defensive wing retraction response (Figure 9). Flies respond to touching of their wings via mechanosensory inputs to interneurons (CTNs) in the metathoracic neuromere that trigger a defensive wing behavior. Since the touch of the wings by male flies is important in courtship, virgin female flies do not display this wing defense response. This is ensured by a pathway where sensory inputs (courtship song and pheromones) to female-specific neurons that express the gene *doublesex* use γ-aminobutyric acid (GABA) to inhibit the CTNs (Figure 9) [118]. In recently mated flies, however, the defensive response is strengthened by activation of the CTNs as part of the post-mating response. This increased activity in CTNs is accomplished as follows. A set of sensory neurons (UNs) in the uterus that express the gustatory receptor Gr32a and the gene *pickpocket* synapse onto ABLKs [118]. The UNs receive signals from the semen and activate the ABLKs, which signal with LK to stimulate the CTNs and thus strengthen the defensive response (Figure 9) [118]. These authors demonstrated that the signal that triggered the UNs was not sex peptide, but rather some other chemical signal in the semen. This suggests that more than one chemical signal can trigger the post-mating response. The link between the action of ABLKs in inducing a post-mating response and their role in regulation of ion and water homeostasis and feeding is not clear at this point. The post-mating modulation is induced by ABLK action within the CNS, whereas the other actions are hormonal. Since recently mated flies display altered activity patterns, feeding and metabolism [134,135,136,137], maybe hormonal LK contributes to this phenotype.

### 3.2. Functions of LHLKs

**Figure 10:**
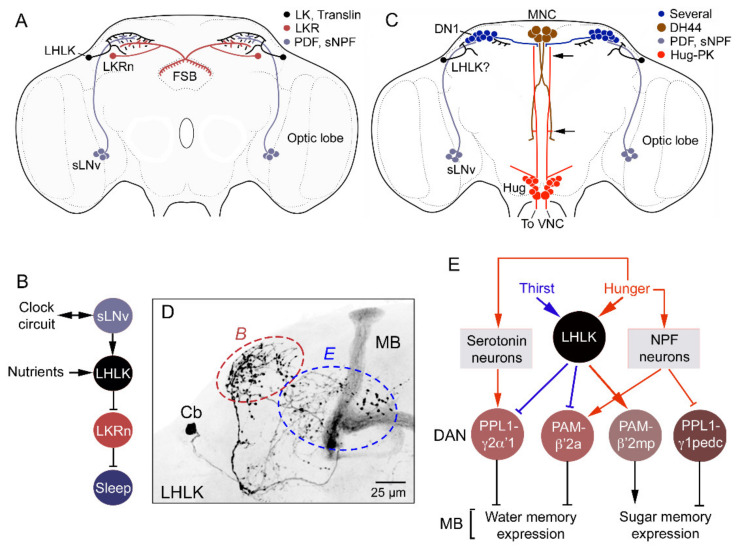
LK signaling in regulation of sleep–metabolism and water memory in *Drosophila*. (**A**) Schematic of brain neurons connecting clock, nutrient sensing and regulation of sleep. Clock neurons (sLNv, small lateral ventral neurons) have outputs on LHLKs that in turn inhibit LK receptor-expressing neurons (LKRn) innervating the fan-shaped body (FSB) and thereby inhibit sleep in a nutrient-dependent fashion. Figure compiled from data in [47,110,111]. Other abbreviations: PDF, pigment-dispersing factor; sNPF, short neuropeptide F. (**B**) A schematic of the connections in A. (**C**) Another sleep-regulating system in the brain with input from sLNv clock neurons (and possible inputs from LHLKs via dorsal clock neurons, DN1). sLNv neurons signal to DN1 clock neurons which, in turn, have outputs on median neurosecretory cells (MNCs) expressing DH44. The MNCs signal via one of the DH44 receptors (DH44R1) to Hugin neurons, which modulate motor circuits in the VNC. Data in this figure are compiled from [139,140]. The possible LHLK input was added here. (**D**) LHLK neuron with arborizations that interact with the circuits shown in panels B and E (red and blue dashed ellipses). (**E**) LHLK neurons and the circuit regulating water–sugar-based memory. The LHLK neurons receive hunger and thirst signals and act on dopaminergic neurons (DAN; PPL1 and PAM subtypes) to regulate expression of water and sugar memory in mushroom body (MB) circuits. Some of these neurons also receive inputs from serotonin and NPF (neuropeptide F) neurons. Redrawn from [116].

**Figure 11:**
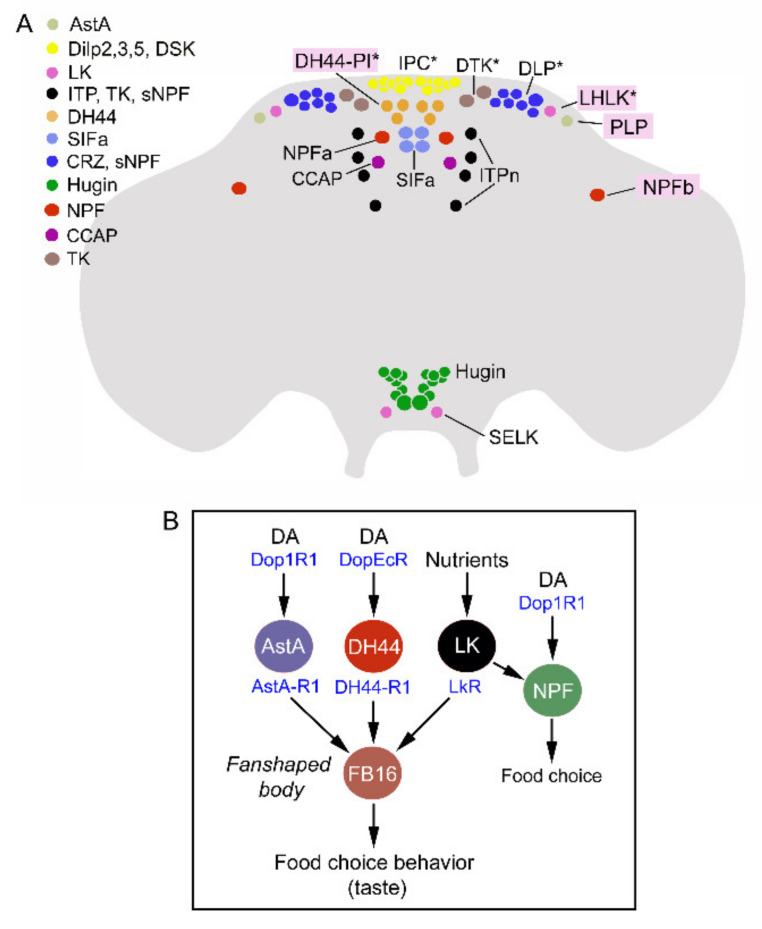
LK neurons and other peptidergic neurons that regulate food choice behavior and feeding. (**A**) Peptidergic neurons and neurosecretory cells that have been implicated in nutrient sensing, food choice and feeding behavior. The neurons highlighted with pink boxes are discussed in Figure 10B. Asterisks indicate that neurons are nutrient sensing. The peptides are as follows: AstA, allatostatin A; DSK, drosulfakinin; ITP, ion transport peptide; TK, tachykinin: sNPF, short neuropeptide F; SIFa, SIFamide; CRZ, corazonin; Hugin, hugin-pyrokinin; NPF, neuropeptide F; CCAP, crustacean cardioactive peptide. Data used for compilation of this figure are from [1,7,141,142,143,144,145]. (**B**) The nutrient-sensitive LHLK neurons (represented by LK) act on neurons (FB16) innervating the fan-shaped body (FSB). Additional neurons converge on the FB16 neurons to regulate food choice: neurons producing allatostatin A (AstA), and diuretic hormone 44 (DH44). Neuropeptide F (NPF) neurons also act on FB16 neurons to regulate food choice. The AstA, DH44 and NPF neurons receive inputs from dopaminergic neurons (DA) via two of the DA receptors, Dop1R1 and DopEcR. This figure was redrawn from [117].

**Figure 12:**
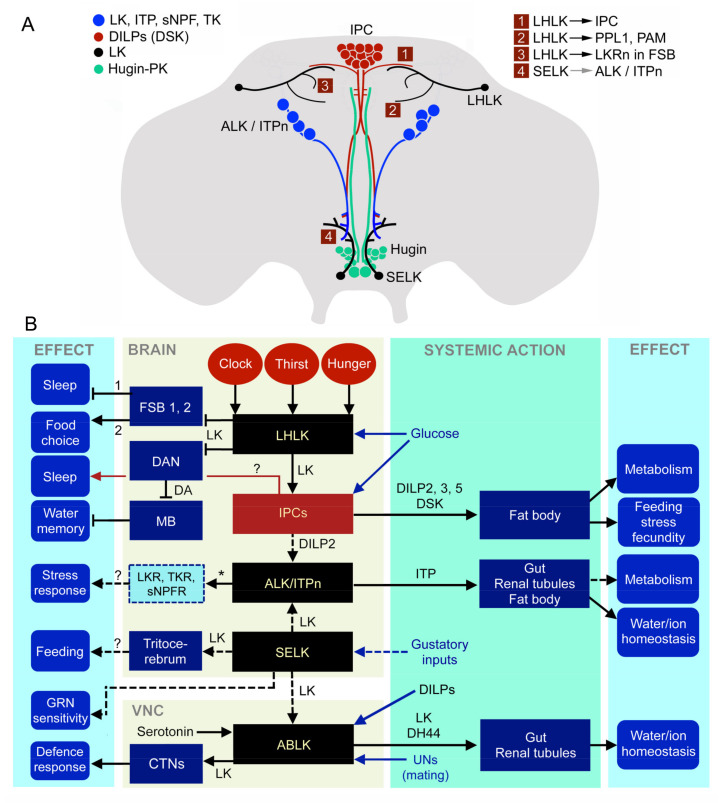
Summary diagrams of leucokinin functions in *Drosophila*. (**A**) Schematic depiction of LK neurons in relation to neurosecretory cells in the adult *Drosophila* brain. LHLKs act on insulin-producing cells (IPCs), dopaminergic neurons (PPL1 and PAM subtypes) and at least two types of LK-receptor expressing neurons (LKRn; FSB 1 and 2 in B) innervating the fan-shaped body (FSB). SELKs may act on the ALK/ITPn that express the LKR. The Hugin neurons of the subesophageal zone are also shown since they form a link between gustatory sensory cells and feeding circuits, including IPCs. The numbered boxes (1–4) indicate sites of interaction between neurons. Data derived from [38,48,110,117]. (**B**) Schematic diagram of functional connections between LK neurons and other neurons, circuits and peripheral targets. Arrows indicate various actions, dashed arrows suggest presumed actions and the stop bars indicate inhibitory action. The LK neurons in the brain and ventral nerve cord (VNC) are shown as black boxes and the insulin-producing cells (IPCs) as a dark red box. Target neurons of LHLKs are shown as dark blue boxes with two types of LKR-expressing neurons of the fan-shaped body (FSB 1, 2; these are LKR neurons and FB16 neurons), dopaminergic neurons (DANs; these are PPL1 and PAM neurons) and mushroom body-associated neurons (MB). LKR neurons inhibit sleep [110] and FB16 neurons regulate food choice [117], whereas MB neurons, via dopamine (DA) inputs, mediate water (and sugar) memory [116]. LHLKs are glucose-sensing and receive inputs from neuronal circuits of the circadian clock and systems sensing thirst and hunger (red ellipses). LHLKs signal with LK to IPCs, which regulates sleep–metabolism interactions [38,48]. IPCs are nutrient-sensing and are known to use DILP2, 3 and 5 to regulate multiple functions, including carbohydrate and lipid metabolism, feeding, stress responses and fecundity (they also express DSK) (see [146]). IPCs are likely to act on ImpL2 expressing ALK/ITPn with DILP2 (dashed arrow) [98]. SELK neurons are likely to receive gustatory inputs [36] and regulate feeding, and their descending axons may act on ABLKs (dashed arrows). They might also provide inputs to ALK/ITPn (dashed arrow). ABLKs release diuretic hormone 44 (DH44) and LK systemically to regulate ion and water homeostasis [37]. ABLKs express serotonin and insulin receptors [100]. In female flies, ABLKs receive inputs from sensory cells in the uterus (UNs). When the UNs are activated by mating, ABLKs act on a set of neurons (CTNs) in the metathoracic ganglion that modulate a circuit that regulates wing retraction as a defense response [118]. ALK/ITPn are neurosecretory cells that use ITP to systemically regulate water homeostasis via the intestine and hindgut and also to regulate feeding and drinking [45]. These cells utilize TK and sNPF to regulate responses to starvation and desiccation [43], but the neuronal circuitry is not yet known. The box with a dashed outline represents neurons expressing receptors for LK, TK and sNPF (and ITP; not shown) in the brain that are yet to be identified. The role of LK in ALK/ITPn cells is not known.

The LK neurons that are most extensively investigated are the LHLKs. As summarized in Table 2 and Figure 10, Figure 11 and Figure 12, the activity of LHLKs is nutrient (glucose)-dependent [48]; they receive inputs from clock neurons [47] and appear to convey the feeding state to different brain circuits such as mushroom body circuits for water and sugar memories [116], the fan-shaped body (FSB) for food choice [117], FSB (and others) for sleep regulation [47,110] and IPCs for feeding and metabolism (and maybe sleep) [38,48]. LHLKs may also (together with SELKs) mediate hunger-regulated nociception [115] and modulate certain olfactory and gustatory signals [114]. Thus, LHLKs are likely to be modulating several neuronal systems in a nutrient- and clock-dependent fashion to regulate state-dependent behaviors.

The two LHLKs have relatively complex arborizations, invading several portions of the dorsolateral brain in one hemisphere where they can interact with specific dopaminergic neurons, FSB, certain clock neurons, IPCs and probably others (Figure 2B,C and Figure 10A–E). Functional imaging using GCaMP6m revealed that application of glucose to the brain reduces Ca^2+^ activity in LHLKs, but not in SELKs [48]. In the same study, it was shown that starvation increases Ca^2+^ activity in LHLKs and refeeding decreases it. These findings suggest that the nutrient level in the circulating hemolymph modulates the activity in LHLKs, but it remains to be demonstrated whether this is by cell-autonomous glucose sensing [48]. In the following, for simplicity, LKLKs are still referred to as nutrient sensing (with the caveat that this could be indirect via inputs from other neurons). Another finding that sheds light on activation of LHLK is that specific knockdown of AMP-activated protein kinase alpha (AMPKα) in LK neurons leads to increased Ca^2+^ activity in the LHLKs of fed flies [48]. This Ca^2+^-response is similar to the effect of starvation, suggesting that AMPK is active in LHLKs in fed flies, and that reduced AMPK signaling during starvation leads to increased LHLK activity [48].

It was shown that peptidergic clock neurons (sLNv) have outputs on LHLKs that in turn inhibit neurons expressing LK receptor which innervate layers of the fan-shaped body (FSB) and thereby regulate behavioral activity and sleep [47] (Figure 10A). Manipulation of neural activity in LHLK neurons furthermore revealed that these neurons are regulating starvation-induced sleep suppression [111]. LHLKs express translin, which is a conserved RNA/DNA binding protein, and this protein is also essential for the starvation-induced sleep suppression [111]. Another study implicated LK in regulation of postprandial sleep [110]. It was shown that LK signaling inhibits this protein meal-induced sleep. As mentioned, the activity of LHLKs is dependent on feeding state and AMPK activity [48]. LHLKs were, furthermore, identified as essential in metabolic regulation of sleep and thus relay nutritional state to the circuits regulating sleep [48]. In the same study, it was found that expression of the LKR in insulin-producing cells (IPCs) was required for sleep regulation in starving flies. Thus, LK signaling from LHLKs to IPCs could be identified as one of several sleep-regulating pathways. Further support for this is that nutritional and circadian inputs to IPCs have been implicated in regulation of activity rhythms in *Drosophila* [138].

As seen in Figure 10C**,** there is another activity/sleep-regulating pathway that connects sLNv neurons to other clock neurons (DN1), which, in turn, act on DH44-expressing MNCs that synapse on descending Hugin neurons in the SEZ to regulate motor activity in the ventral nerve cord [47,127,139]. This pathway may also integrate LHLKs, which arborize in the same region as the sLNv and DN1 clock neurons (Figure 10C), and LHLKs are postsynaptic to sLNvs (Figure 10A), but this circuit has yet to be established.

As mentioned, LHLK neurons are nutrient-sensing, at least indirectly [48], and receive both hunger as well as thirst signals [116]. It was shown that LHLKs act on specific dopaminergic neurons (DANs; PPL1 and PAM subtypes) associated with the circuits of mushroom bodies to regulate expression of water and sugar memory (Figure 10E) [116]. Some of these DANs also receive inputs from serotonin- and NPF (neuropeptide F)-expressing neurons that relay hunger signals.

Another role for LHLKs is in food choice behavior [117]. Quite a number of peptidergic neurons have been implicated in regulation of food choice and feeding (Figure 11A). Among these are the LK-expressing neurons, which interact with a set of neurons (FB16) associated with the fan-shaped body of the central complex (Figure 11B). FB16 neurons are involved in making a final experience-based food choice when flies are exposed to conflicting gustatory stimuli [117]. It was not explicitly tested in the study whether the LK neurons in this circuit are LHLKs, but judging from their anatomical position and their nutrient-sensing capacity, it is quite likely that they are. LK neurons are only one out of several peptidergic systems signaling the FB16 neurons; others signal with allatostatin A, DH44 and NPF (Figure 11A,B) [117]. Upstream of the peptidergic systems (except LHLKs) are dopaminergic neurons acting via two different types of dopamine receptors. The food choices made are determined by integration in the fan-shaped body of taste quality, previous experience and nutritional state of the fly. This suggests a hitherto unknown role for the fan-shaped body in choice encoding and decision-making, based on neural integration of external sensory and internal state inputs [117]. The LHLK functions are summarized in Figure 12A,B.

The complex set of peptidergic neurons/neurosecretory cells that are involved in nutrient sensing, regulation of gustation, food choice, feeding and metabolism, indicated in Figure 11A, suggests many levels of signaling involving several brain circuits. Yet, the two glucose-sensing LHLKs play prominent roles in integrating internal state signals and external cues to influence important circuits involved in choice behavior, nutrient-dependent activity and sleep, as well as certain memory functions. It would be of great interest to investigate the interactions between the LHLKs and the other peptidergic systems depicted in Figure 11A and establish further connections to the circadian clock and sleep-inducing neurons [147,148,149].

### 3.3. Functions of SELKs

SELKs have not yet been specifically investigated. Their location in the SEZ and arborizations there and in the tritocerebrum (Figure 2 and Figure 11) suggest a role at the interface between gustatory sensory inputs and regulation of feeding. In the same region, there is a set of about 20 neurons/neurosecretory cells (Figure 11 and Figure 12A) that express a peptide (hugin-pyrokinin; Hug-PK) encoded by the *Hugin* gene and which integrate gustatory information and motor neurons that control food intake, as well as neurosecretory cells, such as IPCs [22,143,150,151,152]. Thus, this ensemble of 20 peptidergic neurons, some of which are descending neurons, seems to orchestrate global actions related to feeding. SELKs have an anatomical position that is similar, and it may be that they are part of the LK-based regulation of gustatory neurons [114,119] and, perhaps, the control of meal size [39]. The SELK axons that descend throughout the VNC appear, in larvae, to contact the ABLKs posteriorly (Figure 1G,H and Figure 5), and at least one pair of ABLKs express the LKR (Figure 5), suggesting that perhaps the SELKs form a link between LK neurons of the brain and VNC (Figure 12B). Note that adults have not yet been specifically investigated with respect to LKR expression in ABLKs. In summary, no clear experimental data are available to suggest functions of the SELKs, and hopefully, future work will target these neurons.

### 3.4. Functions of ALK/ITPn

The LK signaling role of ALKs/ITPn is enigmatic, since their LK peptide expression is seen primarily in early larvae and the *Lk*-Gal4 expression is also weaker in these cells in adults [36,38]. A similar temporal expression pattern was seen for LK in a set of four primary commissure pioneer (PCP) neurons in the locust brain [153]. These locust neurons were later found to co-express the neuropeptide SIFamide [154]. Since the PCP/SIFa neurons play an important role in axonal navigation during embryonic development of the brain, it may be that LK signaling acts during neuronal path finding and/or differentiation. It remains to be determined whether LK in *Drosophila* ALK/ITPn plays a role during development or whether it is a neuromodulator/neurohormone in larval physiology and behavior (and also in adults under specific conditions).

ALK/ITPn are prominent in the larval brain where the cells express ITP, sNPF and LK [36,43], but their functions are not known at present. The adult ALK/ITPn, in addition, produce TK, and the roles of ITP, TK and sNPF have been investigated in preliminary studies [43,45]. Knockdown of either sNPF or TK in the ALK/ITPn results in decreased survival during desiccation and starvation as well as increased loss of water content after desiccation [43]. This suggests that the neurons are involved in the regulation of water and ion homeostasis (antidiuretic) as well as in the modulation of metabolic and dehydration stress responses. A later study confirmed this by analyzing knockdown and overexpression of ITP in the same neurons [45]. Dehydration of the flies increases the ITP level, and ITP was shown to increase thirst and reduce food intake. ITP also decreases excretion (defecation) and thereby promotes conservation of water. Furthermore, the ITP neurons respond to osmotic and desiccation stress, and disruption of ITP signaling compromises the resistance to these stressors [45], similar to TK and sNPF [43]. Thus, taken together, the three ALK/ITPn peptides studied cooperate to regulate water and ion homeostasis and the ability to cope with ionic and desiccation stress. In addition, ITP promotes thirst, suppresses hunger and represses defecation/excretion [45].

The four pairs of ALK/ITPn express further genes/proteins of interest (Table 1): the LKR [38], the dInR [98], minibrain [99], short neuropeptide F receptor (sNPFR1) [99], Imaginal morphogenesis protein-Late 2 (ImpL2) [98] and activity-regulated cytoskeleton-associated protein 1 (dARC1) [97].

So far, the role of the LKR expression in ALK/ITPn has not been studied, but it may suggest LK signaling to these neurons from SELKs. ImpL2 expression is seen not only in ALK/ITPn but also in adipokinetic hormone (AKH)-producing cells, as well as the Hugin cells of the SEZ, and was shown to promote dInR-mediated insulin signaling (and DILP2 uptake) in these neurons [98]. Minibrain (a tyrosine phosphorylation-regulated kinase) increases sNPF signaling via activation of sirtuin 2 (Sir2; deacetylase) and deacetylation of FOXO (forkhead transcription factor) and promotes food intake [99]. After feeding, protein kinase B (AKT)-mediated insulin signaling suppresses FOXO-induced sNPF expression and food intake is decreased [99]. Thus, ALK/ITPn and sNPF are part of a circuit regulating feeding dependent on nutritional status. Finally, dARC (an activity-regulated cytoskeleton-associated protein) is expressed in insulin-producing cells (IPCs) and ALK/ITPn cells [97]. Flies mutant in dARC display extended survival when exposed to starvation. This starvation resistance may partly be a result of the mutant flies displaying a lack of increased locomotor activity when exposed to starvation [97]. dARC1 was shown to act in IPCs to regulate this starvation-induced behavior, whereas the specific role of ALK/ITPn cells was not studied.

In summary, the functional roles of these intriguing ALK/ITPn cells are far from clear (see Figure 12A,B). It seems that they are at the interface between neuroendocrine systems that regulate water and ion homeostasis, thirst, hunger and metabolism [43,45,97,98,99]. They may even be orchestrating these aspects of physiology and behavior. It is important to note that in the studies cited just above, the specific roles of LK in the ALK/ITPn were not approached, and except for two of the studies [43,45], the analysis was not aimed at these specific neurons. Clearly, further studies of ALK/ITPn cells and their relations to IPCs, AKH-producing cells and other LK neurons will be of great interest. Furthermore, the specific role of LK in these neurons needs to be investigated.

## 4. Lineages of LK Neurons in *Drosophila* and Relations to GPB5 Neurons

**Figure 13:**
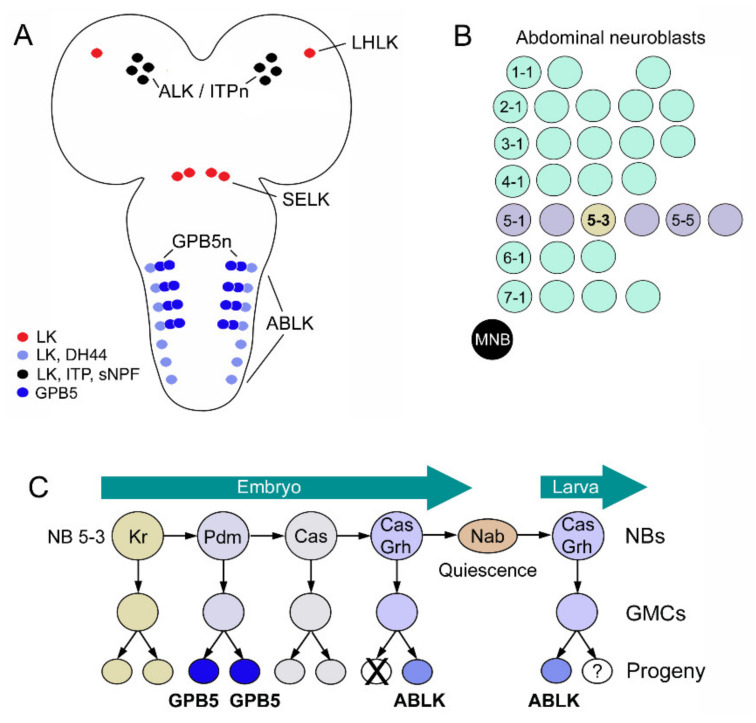
Lineages of LK and GPB5 neurons during development. (**A**) Larval CNS with cell bodies of LK neurons (ALK/ITPn; LHLK, SELK and ABLK) and the peptides expressed in these cells (colored circles). In the first four abdominal neuromeres, there are also neurons expressing the glycoprotein GPB5 (two pairs in each neuromere). (**B**) Neuroblasts in one abdominal hemi-neuromere with rows numbered from 1 to 7. Neuroblast 5-3 gives rise to ABLKs and GPB5 neurons. MNB, median neuroblast. (**C**) Origins of GPB5 neurons and ABLKs from neuroblast 5-3 during embryonic and larval development. Transcription factors: Kr, Krüppel; Pdm, Nubbin/Pdm2; Cas, Castor; Grh, Grainy head; Nab, transcriptional cofactor. This figure (C) was redrawn from [128].

The ABLKs of *Drosophila* have been analyzed with respect to their embryonic neuroblast origin and differentiation throughout development and metamorphosis [128,155]. Interestingly, the ABLKs share ancestry with the neurons producing the protein hormone GPB5 (Figure 13A). Both ABLKs and GPB5 neurons are derived from neuroblast NB5-3 [128] (Figure 13B,C). An earlier study had proposed NB5-5 as the origin of ABLKs [155], but this was corrected in the later study cited above. In a third instar larva, there are eight pairs of GPB5 neurons in abdominal neuromeres A1–A4 and seven pairs of ABLKs in A1–A7. This is accomplished by selective apoptosis of ABLKs in the embryo and a loss of LK phenotype in one daughter cell (progeny) in the larva, whereas all GPB5 neurons remain in A1–A4 (Figure 13C) [128]. These cell fates are determined by a set of transcription factors that are switched on in a temporal sequence (Figure 13C).

LHLKs are derived from brain neuroblast Pcd6 and share most molecular markers (transcription factors) with ABLKs [90,156,157]. One difference is the requirement of apterous in LHLKs, but not in ABLKs [157]. These authors also showed that Notch signaling and Dimmed are important in the specification of the LK phenotypes in ABLKs and LHLKs. It is intriguing that the ABLKs and GPB5 neurons are siblings since they both might be involved in ion and water homeostasis [37,121], even though preliminary analysis of the GPB5 neurons provided no confirmation of this [128]. Future work should address this in more detail and also determine whether the two systems interact in their regulatory roles.

## 5. Some Aspects of LK Signaling in Other Invertebrates

Very few functional studies of LK signaling have been performed outside *Drosophila*. Some early studies employed *ex vivo* analysis of visceral muscle and found that LKs and some stable LK analogs stimulate contractions in the hindgut of *L. maderae* [27,28], the housefly *Musca domestica* [35], as well as the bug *Rhodnius prolixus,* where the midgut was also activated [158,159]. Induction of secretion in Malpighian tubules was shown in *ex vivo* experiments in flies, mosquitoes, crickets, locusts and moths [30,32,33,102,123,160], but not in *R. prolixus,* where LKs may act elsewhere to facilitate diuresis together with DH44 [104,159].

In the mosquito *Aedes aegypti,* LK applied to mouthparts and prolegs inhibits sucrose feeding and *Lkr*-RNAi deletes this LK-induced inhibition [161]. Feeding is also regulated by LKs in *R. prolixus*, ticks and the mollusk *Aplysia californica* [158,162,163]. In *Aplysia*, LK action is within the CNS by neuromodulation in motor pattern-generating circuits in the buccal and cerebral ganglia that control radula muscles during feeding [163]. A recent study showed that in the Asian honeybee *A. cerana*, gustatory responses to sucrose are also modulated by LK signaling [164]. Furthermore, this study showed that the *Lkr* gene influences division of labor in foraging and that nectar foragers display lower *Lkr* expression than those foraging for pollen. In a cattle fever tick, *Lkr*-RNAi delays oviposition and decreases egg numbers and hatchability of eggs laid [162]. Finally, LKs inhibit release of digestive enzymes such as protease and amylase from the midgut in the moth *Opisina arenosella* [165].

Thus, some LK functions appear conserved among several, but not all, insects, such as a role in regulation of gustatory receptors, feeding, water and ion homeostasis and gut function. Clearly, further studies of other insects are required to test whether LK functions discovered in *Drosophila* are more widespread. It can also be noted that the number of neurons expressing LK and the number of LK neuron types vary extensively between insects studied. In fact, *Drosophila* is an example of a strong reduction in both total number and the number of different neuron types, with only 20 neurons in total in the larva (26–28 with the ALKs) of three types (four with the ALKs). In comparison, the cockroach *L. maderae* has 250 neurons of many different types [166].

Another interesting difference between insect species is that the LK precursors can give rise to different numbers of paracopies of LKs. The *Drosophila* precursor encodes one LK [3,4,30], whereas in, for example, the cockroach *Periplaneta americana*, there are 17 [167], and in the western flower trips *Frankliniella occidentalis,* 26 paracopies were predicted [168]. The largest number of paracopies found to date was detected in the sea slug *Aplysia californica*, where the LK precursor may give rise to 60 peptides, of which more than 30 are amidated LKs and the others represent other peptides [163]. It is not clear what the functional consequences are of the expanded number of paracopies, but it may aid in diversification of peptide signaling (neofunctionalization) during evolution if paralleled by GPCR co-evolution [169]. Rarely, it has been shown that sequence-related paracopies display distinct actions. Some work on *Aplysia* has suggested isoform-specific modulatory activities at the neuromuscular junction [170,171]. A different situation is when the precursor gives rise to distinct neuropeptides, some of which may be differentially expressed in neurons due to alternative splicing; in this case, distinct but coordinated peptide actions were shown, for instance, in the mollusks *Aplysia* [172] and *Lymnaea stagnalis* [64]. Peptides different from LKs have been identified on LK precursors in some insect species such as *Frankliniella occidentalis, Rhodnius prolixus* and the bed bug *Cimex lectularius*, but at present, their biological activities are not known [168,173,174]. One aspect of LK distribution is remarkable, with the exception of *R. prolixus* [104]; there are no examples of LK expression in enteroendocrine cells of the intestine. This is in contrast to many other neuropeptides that are found both in the brain and the gut [85,175,176,177]. A separate review deals more extensively with distribution and functional roles of LK signaling in other invertebrates [178].

## 6. Concluding Remarks

This review has summarized the multiple roles of LK signaling in *Drosophila* and emphasized that LK function is based on the action of a very small number of neurons and neurosecretory cells. Including ALK/ITPn, there are four types of LK neurons, three of which reside in the brain/SEZ. The pair of nutrient- and AMPK-dependent LHLKs alone have been implicated in many regulatory functions that link internal nutritional state and inputs from the biological clock to food choice, memory and activity/sleep [47,48,111,116,117] (Figure 12, Table 2). These neurons also act directly on IPCs to modulate their activity and, thus, affect multiple physiological and behavioral aspects of daily life [48] (Figure 12). The mechanisms of LK action in feeding are not completely clear. Possibly, some role is played by nutrient-sensing LHLKs and their action in the fan-shaped body that mediates food choice, but it is also likely that the less-studied SELKs are part of the feeding regulation process. They are located in the SEZ and arborize in that region, as well as in the tritocerebrum, which are neuropils known to receive gustatory inputs and contain interneurons and motoneurons that control feeding (see [22,143,150,152]). ABLKs use LK and DH44 to regulate secretion in Malpighian tubules, and the effect of this on ion and water homeostasis influences flies’ response to starvation, desiccation and ionic stress [37,38]. Since LK is just one out of several peptide hormones involved in the control of ion and water content in the fly, we can expect quite complex interactions between multiple regulatory systems. LK and these other peptide hormones are also likely to be part of a more global orchestration of nutritional- and osmotic state-dependent physiology and behaviors. The recent finding that ABLKs partake in a circuit that changes the behavior of female flies after mating is interesting [118]. The phenotype described in that study is the result of LK acting within circuits of the CNS. It would be intriguing if the ABLKs in these flies also contribute to other aspects of the post-mating response, including activity, fecundity and metabolism [134,135,136,137], by hormonal action of LK. Interestingly, ABLKs seem to be part of different circuits in the larva, where they modulate aspects of locomotion as well as pre-ecdysis behavior and tracheal emptying [40,41,42], and their role in diuresis has not been explored. Thus, ABLKs are versatile and plastic, with roles spanning from larval ecdysis to regulation of several aspects of adult daily life. ABLKs and LHLKs seem to monitor internal states and orchestrate physiology and behavior, and future work is required to establish whether all of the LK neurons function in a cooperative fashion.

## Figures and Tables

**Table 2:** Functions of LK signaling and specific LK neurons in *Drosophila.*

Function	Stage	Cell Type	References
Mediates hunger-regulated nociception	Adult	LHLK, SELK	[115]
Links clock and sleep-regulating neurons (LK inhibits sleep)	Adult	LHLK	[47]
Inhibits postprandial sleep via sleep-regulating neurons (after protein meal)	Adult	LHLK	[110]
Mediates starvation-induced sleep suppression	Adult	LHLK ^1^	[48,111]
Signaling to insulin-producing cells	Adult	LHLK ^1^	[38,48]
Regulation of feeding and metabolism	Adult	LHLK ^1^	[38,48]
State-dependent expression of water- and sugar-seeking memories	Adult	LHLK	[116]
Food choice via circuits in fan-shaped body	Adult	LHLK ^2^	[117]
Regulation of mechanosensory-induced defensive post-mating response in females	Adult	ABLK	[118]
Induces secretion in renal tubules (diuresis)	Adult	ABLK	[30,37]
Water and ion homeostasis, modulation of desiccation response	Adult	ABLK	[37,100]
Larval locomotion	Larva	ABLK	[42]
Pre-ecdysis behavior	Larva	ABLK	[40]
Tracheal clearance at ecdysis	Larva	ABLK	[41]
Regulation of meal size	Adult	Not shown ^3^	[39]
Suppression of feeding	Adult	Not shown ^3^	
Modulation of bitter taste receptor neurons ^4^ in aversive response to bacteria	Adult	Not shown ^3^	[119]
Regulation of starvation-induced hyperactivity	Adult	Not shown ^3^	[120]
Modulation of olfaction and taste responses	Adult	Not shown ^3,5^	[114]
Longevity (LK knockdown extends lifespan)	Adult	Not shown ^3^	[114]

Notes: ^1^ LHLKs are nutrient (glucose) sensing (whether indirectly or directly is not clear) [48]. ^2^ Not explicitly stated in the paper, but likely (see text). ^3^ Not shown, means that general knockdown or activation was used. ^4^ Bitter gustatory receptors Gr66a and Gr33a. ^5^ Presumably LHLK and SELK.

## Data Availability

Not applicable.

## Supplementary Materials

The following are available online at https://www.mdpi.com/1422-0067/22/4/1940/s1. Figure S1. Neurosecretory and efferent neuronal systems in the adult ventral nerve cord that affect ion and water homeostasis.
