# Supplementary material for: Leucokinin and Associated Neuropeptides Regulate Multiple Aspects of Physiology and Behavior in Drosophila
Source: Int J Mol Sci. 2021 Feb 16;22(4):1940. doi: 10.3390/ijms22041940 (PMC7920058; doi:10.3390/ijms22041940)
Supplement: Supplementary file 1 [file ijms-22-01940-s001.pdf]

Supplementary Material

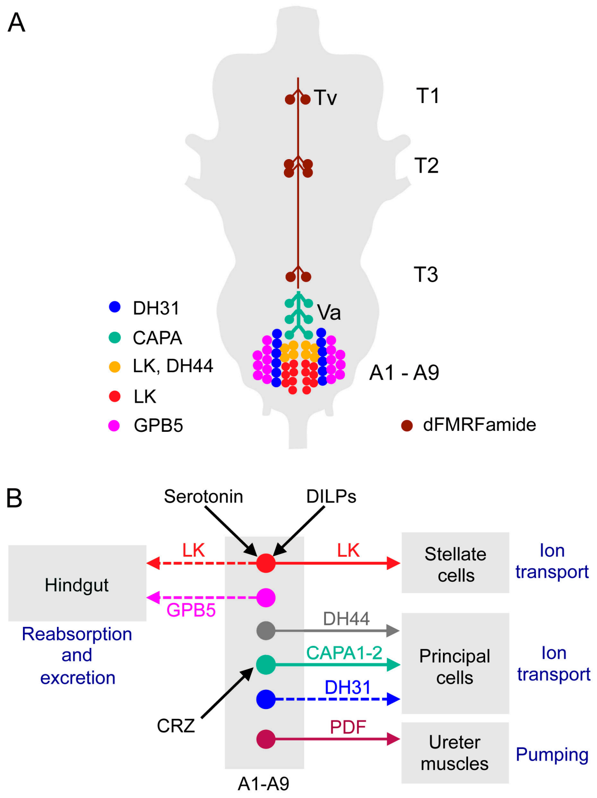

Figure S1. Neurosecretory and efferent neuronal systems in the adult ventral nerve cord that affect ion and water homeostasis. **A.** Cell bodies of neurosecretory cells in the adult ventral nerve cord (VNC) are mainly found in abdominal neuromeres and only a set of FMRFamide-expressing cells is known in the thoracic neuromeres. The peptide acronyms are explained the text. The Va neurons have axon terminations in a neurohemal area in the dorsal neural sheath of the VNC, the others terminate on muscles in the body wall. The Tv cells have axon terminations in a plexus forming a neurohemal area in the dorsal neural sheath of the VNC, the others terminate on muscles in the body wall. dFMRFamide has not been implicated in diuresis; the neurons are shown simply to depict the only know thoracic neurosecretory cells. **B.** Peptide hormones in abdominal neuromeres that regulate water and ion balance. Dashed lines indicate that actions from abdominal cells have not been shown experimentally. Some of the neurons are regulated by specific substances (serotonin, DILPs and CRZ); the LK producing ABLK neurons express receptors for DILPs and serotonin [106] and the Va neurons (CAPA1 and 2) express CRZ receptors [178]. This figure is rearranged from [7].
